# Dynamic Behavior of Sandwich Structures with Magnetorheological Elastomer: A Review

**DOI:** 10.3390/ma14227025

**Published:** 2021-11-19

**Authors:** Umer Sharif, Beibei Sun, Shahid Hussain, Dauda Sh. Ibrahim, Orelaja Oluseyi Adewale, Sumaira Ashraf, Farrukh Bashir

**Affiliations:** 1 School of Mechanical Engineering, Southeast University, Nanjing 211189, China; sidauda.mct@buk.edu.ng (D.S.I.); 233179946@seu.edu.cn (O.O.A.); 2 School of Materials Science and Engineering, Jiangsu University, Zhenjiang 212013, China; shahid@ujs.edu.cn; 3 Institute of Industrial Biotechnology, Government College University, Lahore 54000, Pakistan; dr.sumaira@gcu.edu.pk; 4 Faculty of Basic Sciences, Sardar Bahadur Khan Women′s University, Quetta 87300, Pakistan; neil_kanth@yahoo.com

**Keywords:** magnetorheological materials, magnetorheological elastomers, sandwich structures, dynamic behavior, vibration control

## Abstract

Magnetorheological (MR) materials are classified as smart materials that can alter their rheological features once exposed to peripheral magnetic fields. MR materials have been a standard and one of the primary smart materials for the last few decades due to their outstanding vibration control performance in adaptive sandwich structures and systems. This paper reviews the vibration suppression investigations of flexible constructions using MR elastomers (MREs). In relations of field-dependent controllability, physical features such as stiffness and the damping of different geometrical structures integrated with the core layer of MREs are explored. The veracity of the knowledge is discussed in this article, whereby sandwich structures with different MR treatment configurations are analyzed for free and forced vibration, MRE sandwich structures are analyzed for stability under different working conditions, and the optimal positions of fully and partially treated MRE sandwich structures for improved vibration control are identified. MR materials′ field-dependent stiffness and damping characteristics are also discussed in this article. A few of the most noteworthy research articles over the last several years have been summarized.

## 1. Introduction

Sandwich structures are frequently employed in the design and fabrication of lightweight structural systems due to their ability to provide superior structural and thermal performance while requiring less material [1,2]. Such structures have been utilized as load-carrying members with a high strength-to-weight ratio in structural engineering for many years, such as airplanes, military aircraft, space vehicles, bridges, ships, surface transport vehicles, and robotics [3,4,5]. Passive damping treatments have traditionally been used effectively on numerous structures to reduce vibration response and eradicate vibration-induced noise. Smart materials have recently demonstrated appealing potential for designing adaptive sandwich structures that can effectively dampen vibrations over a wide frequency range [6].

Magneto-rheological (MR) materials are smart materials whose rheological characteristics can be controlled by applying magnetic field externally [7]. MR materials can be classified as magneto-rheological plastomers (MRPs), magneto-rheological polymer gels (MRPG), magneto-rheological grease (MRG), magnetorheological fluid (MRF), and magneto-rheological elastomer (MRE), based on their mixing components [8]. MRF has gained the most popularity among all MR materials due to its rapid response, affected rheology variation, and simple preparation technique. Sedimentation is a limitation of the MRF caused by a discrete density divergence between magnetic particles such as carbonyl iron (CI) power particles and carter oil. To address this issue, innovative MR materials with several types of carter mediums have been suggested. MRE, MRG, MRPG, MR foam, and MRP are the acronyms for these.

MRE is a kind of rubber-like solid material with a rubber or elastomer matrix and is also recognized as a magneto-sensitive elastomer. There are polarized magnetic particles, a natural or synthetic rubber matrix, and additives such as silicon oil [9]. A non-magnetic solid or gel-like matrix surrounds the micron-sized magnetic particles in MRE, which helps overcome difficulties such as particle mismatch and a reduction in the strength of the MR effect exhibited by MRFs. Magnetic particle dispersion in the MRE can be either homogenous (isotropic) or columnar in chain-like form (anisotropic) depending on the cure process under no magnetic field or magnetic field, respectively [10].

**Figure 1:**
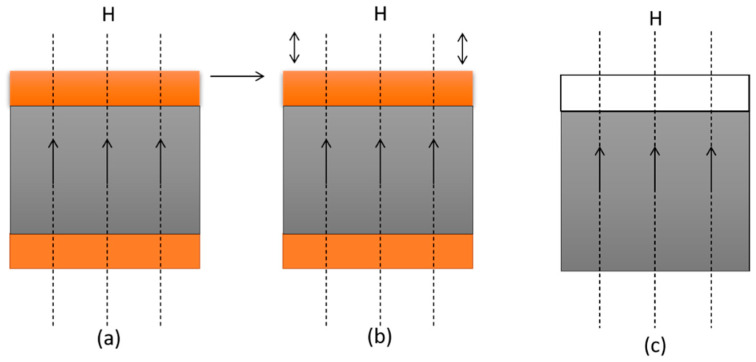
Representation of operational modes of MRE: (**a**) shear mode, (**b**) squeeze mode and (**c**) field-active mode.

There are many analogical mechanical behaviors, but MRE materials have one unique mechanical behavior: they have a field-dependent modulus in the pre-yield region, unlike MRF and MR foam, which have an MR effect in the post-yield region, which makes MREs more stable and extensively applicable in the semi-active control of flexible structures. According to research [11], the maximum upsurge in the modulus of MRE is 40% higher than the preliminary modulus, which is nearly 0.6 MPa. However, the use of MRE has numerous advantages over the use of other MR materials, including the capability to work in multi-degree of freedoms and in applications where variation in stiffness and frequency is required as well as the elimination of the requirement to rearrange magnetic particles in the existence of a magnetic field [12]. Magnetic particles are trapped inside the polymeric or rubber matrix and can be excited externally. As a result, the particles can only move in their defined directions and do not coincide with a magnetic field. The magneto-active property of MREs, however, distorts the magnetic field in soft elastic materials. These characteristics distinguish MRE′s operational mode from MRF′s. Figure 1 depicts the operational modes of MRE. MRE′s operational modes are identical to MRF′s, with the exception of the flow mode, which does not apply to MRE. Shear mode, squeeze mode, and field-active mode are the three basic operational modes that apply to both isotropic and anisotropic MREs. Nonetheless, MREs can alter their shape in field-active operational mode, e.g., when effected by a magnetic field, a property acknowledged as magnetostriction [13].

Previous studies have looked at the applications of MREs in ATVAs [10,14,15,16,17,18,19,20,21], vibration isolators [22,23,24,25], adaptive base isolators [26,27,28,29], stiffness tunable suspensions and mounts [30]. Some investigations on material empirical modeling [31,32,33,34,35,36,37,38,39,40], material development and property testing [41,42,43,44,45,46,47], innovative device design and characterization [48,49,50], performance evaluation [51,52,53,54,55,56], and sandwich beams [57,58,59,60,61] have also been presented, but few looked at the dynamic analysis of MRE sandwich structures. Because the sandwich structure has a high rigidity-to-weight ratio, it is easy to combine and optimize different surface materials as well as the core layer material. The MRE sandwich structure not only achieves variable stiffness and damping, but also ensures the rigidity of the composite structure and the ability to resist deformation while taking vibration and noise reduction into account. As a result, to address the rapidly growing demand and to identify the research challenges in this field, this paper attempts to provide a timely review of the dynamic characteristics of sandwich structures with an MRE core, with a particular emphasis on constitutive models of materials, theoretical modeling and analysis methods, and experimental testing techniques for vibration and damping analysis. Additionally, the final section discusses the effect of various parameters such as the applied magnetic field, boundary conditions, geometry, nanoparticles, and the optimal location of the MRE patch on the vibration, damping, and stiffness of MRE-cored sandwich structures, as well as the nonlinear behavior of MRE-based sandwich structures and systems. This review article is certain to provide extremely useful guidelines for the fabrication of MRE-based sandwich structures, as well as accelerate the development of more innovative research activities and opportunities for future research in the area.

## 2. Characterization of Magnetorheological Elastomer Material

An elastic plastic material model can be used to explain the rheological properties of MRE material. There have been numerous models devised to determine the moduli of storage and loss of MRE in relation to induced field and frequency. It is worthwhile to note that no complete model of these smart materials′ pre-yield behavior has been created. Due to the complex constitutive relationship, different models should be utilized to explain the rheological characteristics of MR materials. The most generally used models to evaluate the rheological characteristics of the smart elastomer in the pre-yield area are viscoelastic models. The dynamic constitutive equation of viscoelastic materials has a noteworthy impact on the dynamic analysis of elastic–viscoelastic complex structures. Some typical viscoelastic models are thoroughly described.

### 2.1. The Basic Form of Constitutive Equations

Suppose the viscoelastic material is linear, isothermal, homogeneous, and isotropic, and it can be expressed in the form of a Boltzmann solid integral [62]. that is:

(1) $$\sigma \;\left(x,t\right)=\chi \left(t,\tau \right)\varepsilon \left(t\right)+\overset{t}{\underset{0}{\int }}\partial \chi \left(t,\tau \right)\;/\partial \tau )\varepsilon \left(\tau \right)\;d\left(\tau \right)$$

where t is time, σ (x,t) is stress, ε(t) is strain, χ( t,τ)= E(τ)+ Q(t,τ)  is a function about τ (t ≤ 0), E(τ) is Young′s modulus and Q(t,τ) is the relaxation function, if t < 0, Q(t,τ)=0, ε(t)=0. The relaxation modulus is generally a continuous monotone non-increasing function because of the characteristic of “attenuation forgets” of viscoelastic materials.

For non-aging viscoelastic materials, the Equation (1) can be simplified as:

(2) $$\sigma \;(x\text{,}t)=\int_{0}^{T}\;\mu (t\text{,}\tau )\;\varepsilon (\tau )\;d(\tau )$$

where:

(3) $$\mu (t\text{,}\tau )=\chi (t\text{,}\tau )\;\varepsilon (t-\tau )-\frac{d}{d\tau }\chi (t\text{,}\tau )=-d/d\tau [H\;(t-\tau )\;\chi (t\text{,}\tau )]$$

H(t) is unit step function.

Fourier transformation is applied to Equation (2), and the result is:

(4) $$\;\hat{\sigma }(\omega )\;=\;\hat{u}(\omega )\hat{\varepsilon }(\omega )$$

Sign plus “^” indicates the Fourier transform of corresponding variable, and $\hat{u}$(ω) is complex modulus of viscoelastic materials.

### 2.2. Mathematical Representation of The Storage and Moduli

Under the action of harmonic external force, the strain of the viscoelastic material lags behind the stress, and the stress–strain curve is a hysteretic loop. Suppose $\hat{u}$(ω) = G*(ω)

G*(ω) = G′(ω) + iG″(ω)(5)

where G′(ω) and G″(ω) are the storage modulus and loss modulus of viscoelastic materials, respectively [63].

(6) $$G^{\prime }(\omega )=x\;\left(\infty \right)+\omega \int_{0}^{\infty }x\;\left(t\right)-\;x\;\left(\infty \right)\;\sin\omega \mathrm{tdt}$$

(7) $$G^{\prime \prime }(\omega )=\omega \int_{0}^{\infty }\left[x\;\left(t\right)-\;x\;\left(\infty \right)\right]\;\cos\omega \mathrm{tdt}$$

With the application of the complex modulus model, in the steady-state harmonic vibration, the viscoelastic sandwich structure forced vibration differential equation can be written as the complex form:

(8) $$[M]\;\{\ddot{q}\}+(1+i\eta )\;[K]\{q\}=\left(f_{0}\right)e^{i\Omega t}$$

That is:

(9) $$[M]\;\{\ddot{q}\}+[K*\;]\{q\}=\left(f_{0}\right)e^{i\Omega t}$$

Thus, we can use the complex arithmetic to find the solution of the complex form, where η is the damping loss factor of structure; [M] and [K*] are the mass matrix and stiffness matrix of the system, correspondingly; {q} is displacement matrix; and $f_{0}$ is coefficient matrix of the excitation.

The basic characteristics of the complex modulus model are non-frequency variation and simple form, which can better describe the mechanical properties of viscoelastic materials under the harmonic excitation. At present, much of the literature adopts complex constant models.

Actually, the viscoelastic modulus of the material is frequency dependent, so complex constant model is only applicable to the analysis of steady-state harmonic vibration in finite frequency ranges.

### 2.3. General Linear Viscoelastic Model

Weiss et al. [64] theoretically studied the rheological properties of MR materials. The results show the stress–strain relationship of MR material divided into three regions (pre-yield region, yield region, and post-yield region); in the pre-yield region, MR materials′ performance depending on the viscoelastic properties. The linear viscoelastic theory can be utilized to explain the viscoelastic properties, that is:

τ = G* γ                  τ<τ_y_ (10)

Here τ represents the shear stress of the material, γ is shear strain of the material, τ_y_ is yield strength, and the complex shear modulus G* is:

(11) $$G*(B)=G^{\prime }(B)+iG^{\prime \prime }(B)$$

Here G′ is the storage modulus, which is proportionate to the average value of the energy stored in material in the unit volume in a certain deformation; G″ is the loss modulus, which is proportional to the energy consumption of material in the unit volume after a period of deformation.

The ratio between the storage modulus and the loss modulus is called the loss factor of the material, that is:

η(ω) = G″(ω)/G′(ω)(12)

where ω is the external excitation frequency when material withstands the shear deformation.

Qing Sun et al. obtained a set of data through experimentation, and the nonlinear relationship between the complex modulus and magnetic field strength of the magneto rheological material was fitted [65]:

G′(B) = 3.11 × 10^−7^ B^2^ + 3.56 × 10^−4^ B + 5.78 × 10^−1^ (13)

**Figure 2:**
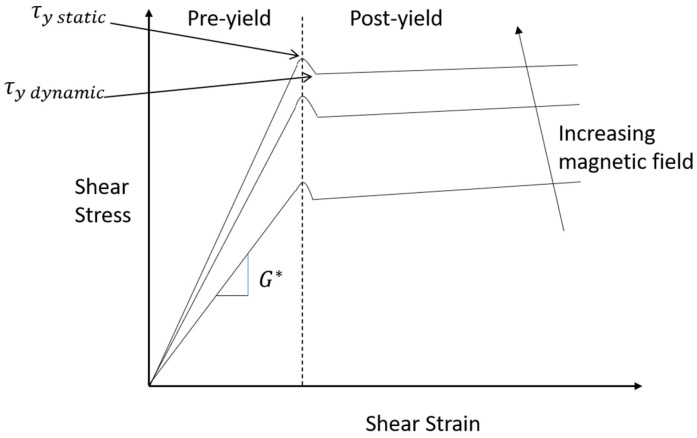
Shear stress–strain relationship of MR material.

G″(B) = 3.47 × 10^−9^ B^2^ + 3.85 × 10^−6^ B + 6.31 × 10^−3^ (14)

where B is magnetic flux density (unit: Gauss). The research showed that the shear stress of the MR sandwich beam is relatively small, and the shear strain of the MR layer is less than 0.1%; thus MRE materials work in the pre-yield region. So, the stress–strain relationship of the MR materials can be defined by linear viscoelastic model. In addition, Ginder provided the relationship between the shear modulus and the applied magnetic field on MRF [66], which is presented in Figure 2 below, and the relationship is as follows:

(15) $$G^{\prime }(B)=3\phi u_{0}M_{s}B$$

where ϕ is the volume fraction of the suspended particles (such as ferromagnetic particles) of MR materials, $u_{0}$ is permeability of free space, M_S_ is saturation magnetization of ferromagnetic particles, and B is magnetic flux density.

### 2.4. Pre-Yield MRE Rheological Characteristics Represented by Viscoelastic Models

**Figure 3:**
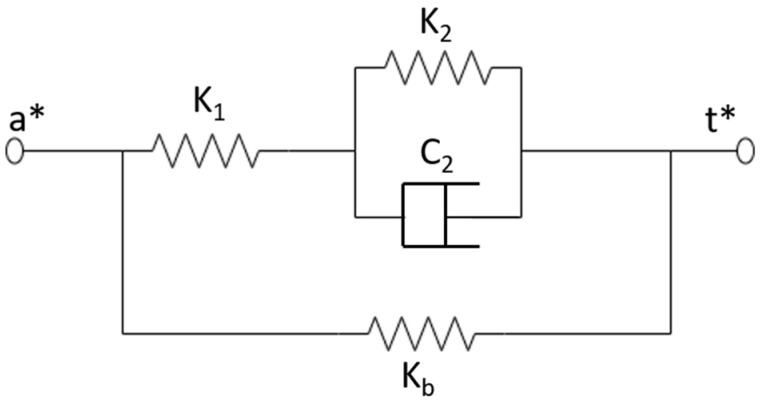
Four-parameter Viscoelastic Model.

The standard rheological model is more accurate and has been widely used in structural dynamic analysis and other aspects [67]. There are two main kinds of analysis models to describe the MR elastomers: the Maxwell model and the Kevin–Voigt model [68,69]. However, with the Maxwell model, it is difficult to analyze the creep behavior of the material, and the Kelvin–Voigt model lacks description of the stress relaxation behavior. Li et al. extended the Kelvin–Voigt model and proposed a four-parameter viscoelastic model to describe the MR elastomers. The model can better describe the viscoelastic behavior of MR elastomers. The specific model is shown in Figure 3. [70] Furthermore, Li used the least square theory and the optimization toolbox of MATLAB to estimate the value of the parameters and made a comparison between the result and the experimental data shown in Table 1.

## 3. Modeling of MRE Sandwich Structures

**Figure 4:**
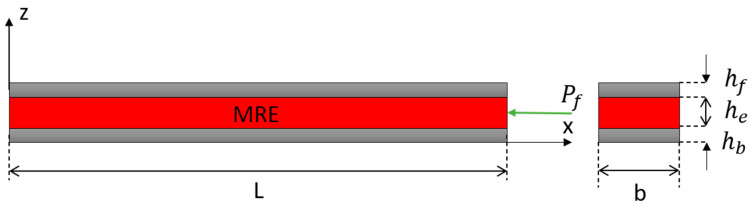
Sandwich beam with MRE core.

Given MRE′s viscoelastic behavior, all models illustrating the vibration features of viscoelastic sandwich structures are possibly relevant to MRE adaptable structures in the pre-yield zone. Ross et al. [71] developed the first model of a viscoelastically damped structure in shear configuration, which is recognized as the Ross–Kerwin–Ungar model (RKU). David investigated the RKU model and discovered that it is constructed on a modified Euler–Bernoulli beam equation and is stated as:

(16) $$m\left(x\right)\frac{\partial^{2}w}{\partial t^{2}}+\frac{\partial^{2}}{\partial x^{2}}\left(\mathrm{EI}\frac{\partial^{2}w}{\partial x^{2}}\right)=0$$

where m(x) is the beam′s mass per unit length and EI is the flexural rigidity of structure. Coulter and Duclos first employed the RKU model to the MRE sandwich beam with a simply supported structure, and through rheological experiment, they obtained related data of the complex shear modulus [72]. The results indicate that the theoretical RKU model′s calculation results correspond well with the experimental results. The schematic diagram of the sandwich beam with MRE is presented in Figure 4.

However, the calculation amount of the RKU model is too large, restricting its application. So, in practical application, the finite element method (FEM) is the most extensively stated method in dynamic investigation of MRE sandwich structures. The finite element method is a more accurate approximation method, which can transform a continuous unlimited freedom problem into a discrete finite degree of freedom problem. There are too many analyses and experiments that show that the finite element method is an effective method which cannot be replaced. Jacques introduced a numerical analysis of the nonlinear vibration characteristics of a viscoelastic sandwich beam by using FEM [73]. Another application of the finite element method was used by Nayak in a rotating sandwich with an MRE core [74]. The governing equation of motion is expressed in a matrix form:

(17) $$\left[m]\;\{\ddot{x}\}+[k]\;\{x\}=\{f\right\}$$

where [m], [k], and {f} are the mass matrix, stiffness matrix, and force vector, respectively. Besides, the Hamilton energy method and Ritz method have also been widely reported. Sun [65] developed governing equations of motion of MR-based sandwich beams by using the Hamilton principle, as follow:

(18) $$\rho \frac{\delta^{2}w}{\delta t^{2}}+2E_{f}I_{f}\frac{\delta^{4}\omega }{\delta x^{4}}-G^{*}bh_{2}\left(\frac{\lim}{\delta x^{2}}-\frac{\delta \varphi }{\delta \chi }\right)=f\left(x;t\right)$$

where ρ is the beam density, E_f_ is Young′s modulus of respective surface/face layer, $G^{*}$ is the complex shear modulus of the core layer, and I_f_ is the moment of inertia at the elastic layer′s centroid. Additionally, b is the beam width, and *h*_2_ is the core layer thickness. Nayak investigated the dynamic characteristics of the MRE sandwich beam and developed the equation of vibration characteristics by using the extended Hamilton energy method. At the same time, the influences of parameters such as the magnetic field strength, the ratio of the ferromagnetic material, the length of the sandwich beam, and the center layer thickness of the magneto rheological elastomer on the natural frequency of the system were analyzed [75]. Aguib et al. researched the dynamic behavior of the magnetorheological elastomer sandwich plate using the Ritz method [76]. The Ritz method uses the basis function to describe the displacement of the structure, and the structure matches the geometry boundary conditions. The governing equation expressed in this method is as follow:

(K − ω^2^M) C = 0 (19)

where C is the vector of the arbitrary coefficient, expressing the displacement field, K is the stiffness matrix, and M is the mass matrix. The normal stress in the core layer, the shear strain, and stress components in the elastic layers are neglected in all of these methods, and there is no slipping between the elastic layers and viscoelastic layer.

## 4. Experiment Methods on MRE Sandwich Structures

The theoretical analysis methods of the calculation and modeling of the characteristics of the MRE and the dynamic characteristics of the sandwich structure are mostly carried out by the simplified object. However, it is necessary to conduct the experiment to verify the theoretical results. In the experimental study of the vibration characteristics of the MRE, the fabrication of the MRE is very important. The fabrication of MRE first appeared in 1995 in Shiga, from the Research and Development Laboratory of Japan′s TOYOTA center, which fabricated the prototype of MRE, a kind of magnetic gel mixed with silicone rubber and iron powder. In the fabrication process, typically the natural rubber and synthetic rubber as the elastic matrix, silicone rubber is the most common matrix. Jolly developed MRE based on silicone rubber, and the results show that the shear modulus of MRE in an applied magnetic field increases about 40% more than the original [11]. Because the mechanical properties of silicone rubber are relatively poor, a lot of researchers have used other rubber as the matrix. Hu fabricated the MRE by mixing polyurethane with silicone rubber, and the test results exhibit that the MRE has a higher magnetic rheological effect [77].

**Figure 5:**
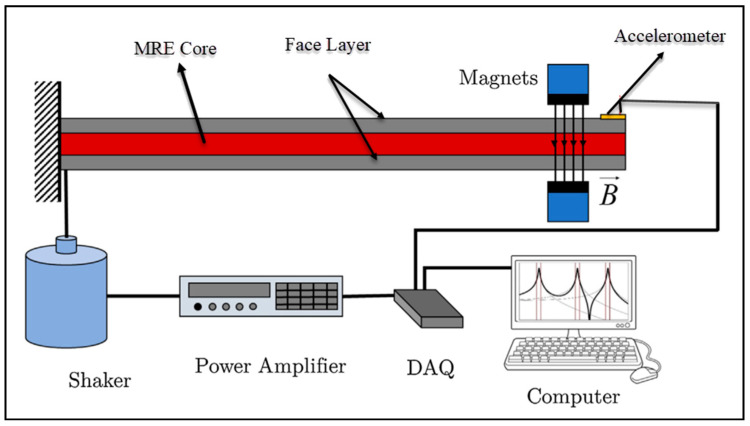
Schematic typical experimental setup of MRE sandwich beam.

In most of the experimental studies, the elastic surface layer of the MRE sandwich beam is made of aluminum alloy material, because the aluminum alloy has lower damping properties and higher stiffness compared with MRE. Moreover, the relative permeability of aluminum alloy is almost 1, so the size and distribution of the applied magnetic field on the MRE will not be disturbed. Free vibration, impact test, and forced excitation are the main experiments that have been conducted in studies. Experimental methods include the experimental modal analysis technique, in which the main principle is using the real or mechanical structure model and the product as the object to execute dynamic experiments, data acquisition, signal analysis and processing, frequency response function estimation of response signal of test points of the exciting force and structure, and then dynamic modal parameter identification (curve fitting), which can achieve quite a high accuracy of modal frequencies, modal shapes and modal damping. The typical experimental setup is shown in Figure 5.

## 5. Observations and Findings

The theoretical analysis and experimental studies of MRE are mainly focused on the effects of various parameters on the dynamic responses of the MRE sandwich beam. Most of the research studied the effect of an applied magnetic field on the frequency response, loss and storage moduli of the structure. However, some studies have reported the effect of the geometry, partial treatment of MRE, and nonlinear behavior of MRE on the dynamic characteristics of sandwich beams consisting of an MRE core layer. 

### 5.1. The Influence of The Applied Field on The Natural Frequencies of MRE Sandwich Structures

The magnetic/electric field increases as the natural frequencies of the structures increase. This effect, which has been seen in numerous investigations, is caused by an increase in the MRE′s complex shear modulus as the applied field is increased. Several investigations, however, found that when the applied magnetic field rose, the natural frequencies of MR-based sandwich beam structures decreased.

A hybrid magnetic system and a self-sensing laminated MRE structure were used to build and test a self-sensing MRE vibration absorber in a study by Sun et al. [78]. The findings of the experiments showed that the suggested absorber′s natural frequency changed from 8.5 Hz at 0 A to 4.8 Hz at 3 A and 11.3 Hz at 3 A. Compared to a passive MRE absorber, the suggested self-sensing MRE absorber improved vibration controllability.

**Figure 6:**
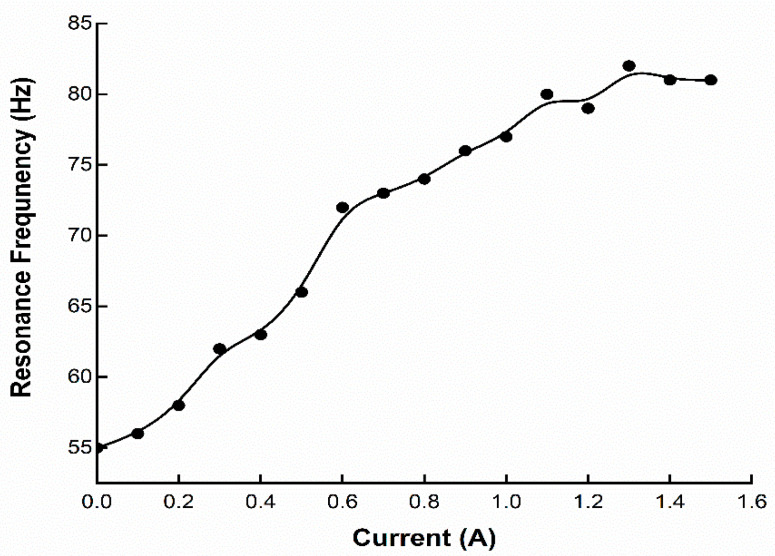
Resonance frequency response of ATVA under different magnetic fields.

According to Deng et al. [79], a magnetic field influenced the modulus of an ATVA made of MREs. At two different end-supported circumstances, the natural frequencies of the ATVA were theoretically studied and experimentally confirmed. The frequency shift of the developed ATVA can be seen in Figure 6, which surpasses the traditional passive absorber.

Sun et al. [80] created an ATVA consisting of a multilayer MRE. They demonstrated that by applying a 3.5 A electric current can alter the tuning frequency from 13.5 Hz to 19 Hz for the ATVA with the multilayered MRE. ATVA made of MRG that could alter the natural frequency from 56 Hz to 67 Hz under the influence of a 100 mT magnetic field was proposed by Kim et al. [81]. Later, the frequency shift feature of the MRE-based vibration absorber used in powertrain mount systems was examined by Xin et al. [82]. An increase from 0 to 2 A in the applied current caused the shift in the natural frequency from 22.7 Hz to 31.9 Hz. Liu et al. [83] developed an absorber made of MRE for propulsion shaft systems, demonstrating that applying a 5A current caused a shift from 43.09 Hz to 56.88 Hz in the natural frequency. Jang et al. [84] used MRE to create a compact TVA. Experimental tests in this study revealed that a magnetic field of 340 mT may shift the tuning frequency of the planned TVA from 51.6 to 71.9 Hz. An MRE-based absorber with an eccentric mass and multilayer MRE design was designed by Yang et al. [85]. The translational frequency tunes were below 2 A of current from 6.99 to 9.66 Hz, while the rotational natural frequency tunes were between 3.51 Hz and 4.45 Hz. ATR 43 and 74 airplane fuselages were tested by Lerner et al. [86] using three distinct MRE vibration absorbers (shear device, longitudinal device, and squeeze model device). There was a shear mode and a squeeze/elongation mode for each of the three devices, and the C-shaped magnetic circuit was used for all three. The natural frequency of all three MRE devices increased by 183 percent for the shear device, 473 percent for the longitudinal device, and 507 percent for the squeeze mode device when the field strength was between 0 and 183 kA/m. Recently, Komatsuzaki et al. [87] created a self-sensing MRE-based dynamic vibration absorber. The external magnetic field can influence the elastomer′s natural frequency and electrical resistance. The system tuning frequency in 316 mT magnetic field was determined to fluctuate from 25.8 Hz up to 37.4 Hz. Li and Zhang [88] designed a damper assembly based on MRE to reduce the vibration of a driveline. This damper improved the system′s ability to transmit power from the engine to the drive wheels, and it can be adapted to different frequencies by using this assembly.

**Figure 7:**
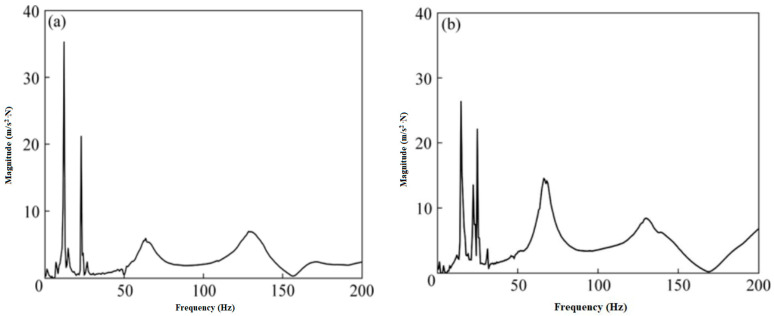
Vibration amplitude vs. frequency response: (**a**) without magnetic field and (**b**) with magnetic field [89].

**Figure 8:**
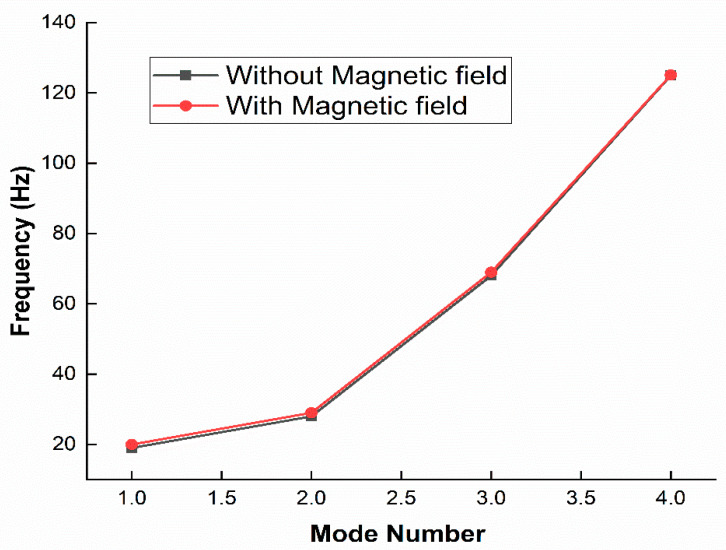
Natural frequency response with and without magnetic field.

Ke-Xiang et al. [89] investigated the forced vibration responses of an aluminum beam with an MRE core. It was found that raising the strength of the magnetic field boosts natural frequencies while reducing vibration amplitude, as shown in Figure 7 and Figure 8.

**Figure 9:**
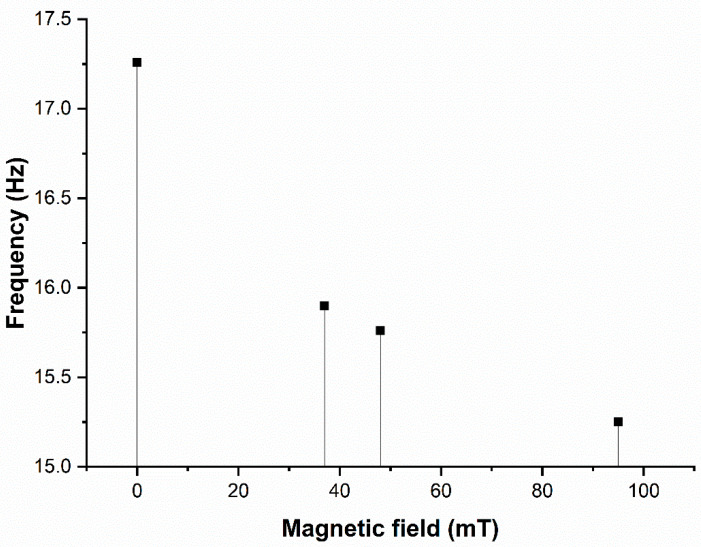
Experimental natural frequency at different magnetic field regions.

Hu et al. [90] studied the vibration properties of the MRE sandwich beam in a nonhomogeneous magnetic field. The existence of a nonhomogeneous magnetic field reduced the initial natural frequency by 13.9 percent of the MRE sandwich beam, as presented in Figure 9.

**Figure 10:**
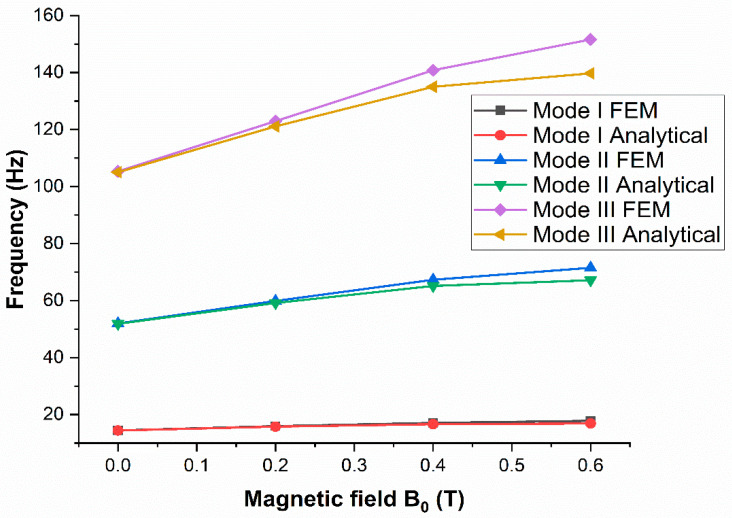
Frequency response under different magnetic intensities.

Nayak et al. [74] investigated the dynamic stability of a rotating MRE sandwich beam when subjected to an axial periodic force. The magnetic fields, rotation speed, hub radius, setting angle, and static load factor all had an effect on the natural frequencies and loss factors of MRE sandwich beams, as shown in Figure 10.

**Figure 11:**
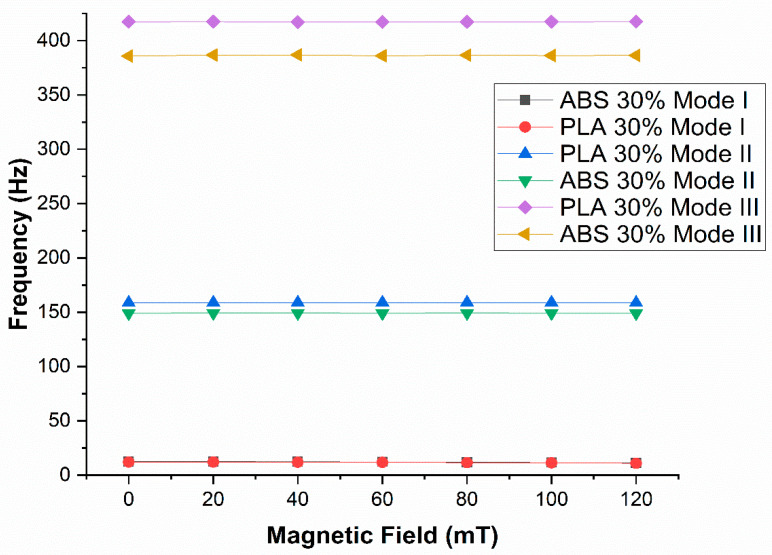
Variation of the natural frequency for different modes considering the magnetic field applied on sandwich structure with MRE honeycomb core of ABS and PLA honeycomb.

Kozlowska et al. [91] used an experimental study to determine the free vibration performance of a carbon fiber-reinforced polymer (CFRP)/MRE sandwich beam. SEM and rheometer were used to examine the microstructure and rheological characteristics of MRE and CFRP/MRE samples. Using CFRP/MRE sandwich beams, the impact of a non-homogeneous magnetic field, amplitude, and logarithmic decline was investigated. They concluded that MRE reduced vibration amplitude and improved damping in the CFRP/MRE structure due to its stiffening effect. Eloy et al. [92] investigated sandwich panels with CFRP skins and an MRE honeycomb core, which exhibited both free and forced vibrations. They concluded that the vibration amplitude of the sandwich panel reduced the magnetic field on the free end of structure was increased, and the downside shifted in natural frequencies [93], as shown in Figure 11. The numerical analysis of a sandwich beam with a hexagonal honeycomb of different materials and an MRE core was conducted by Sharif et al. [94,95], and the down shift in natural frequency was observed for the first three modes for all the structures when the magnetic field was increased.

### 5.2. Effect of The Applied Field on MRE Sandwich Structures Loss Factors and Deflection

In terms of loss factor, the damping properties of adaptable sandwich structures have received a lot of attention. The ratio of imaginary-to-real components of complex eigenvalues is defined as the loss factor. The viscoelastic behavior of MRE composed of RTV silicone rubber and carbonyl iron powder (CIP) was studied by Bellan and Bossis [96]. The applied magnetic field conditions were discovered to have an effect on the shear storage and shear loss modulus. Gong et al. [97] examined the rheological characteristics and microstructures of several isotropic MRE sample assemblies. MRE samples are made up of CIP, silicon rubber, and silicone oil in varying weight percentages. Experiments indicated that mixtures comprising 60% CIP, 20% silicone oil, and 100% silicone rubber have the best MR effect. Chen et al. [98] investigated the shear characteristics of anisotropic MREs and observed that the shear modulus of MREs increases as magnetic fields increase.

**Figure 12:**
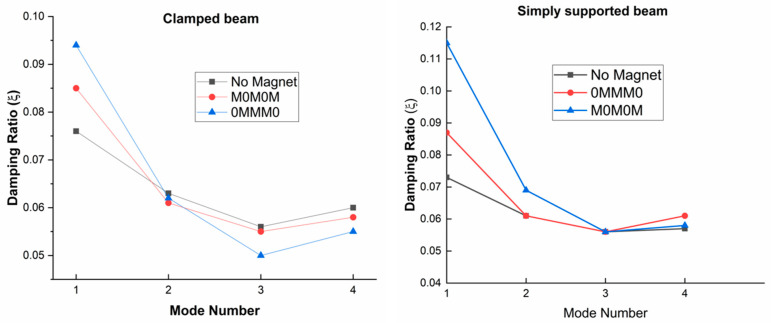
Effect of magnets on damping ratio placed at different positions for simply supported and clamped beam.

The higher-order sandwich beam theory was used by Choi et al. [99] to analyze the dynamic response of a sandwich beam with face skins of steel and an MRE core layer. Experiments using natural frequencies verified the numerical results. The Figure 12 demonstrates the effect on the damping of MRE sandwich structures by applied magnetic fields.

Wang et al. [100] examined the transient vibration response of primary systems attached to MRE-based dynamic vibration absorbers under impulse excitation. By adjusting the stiffness of the vibration absorber, the system′s transient vibration was reduced.

**Figure 13:**
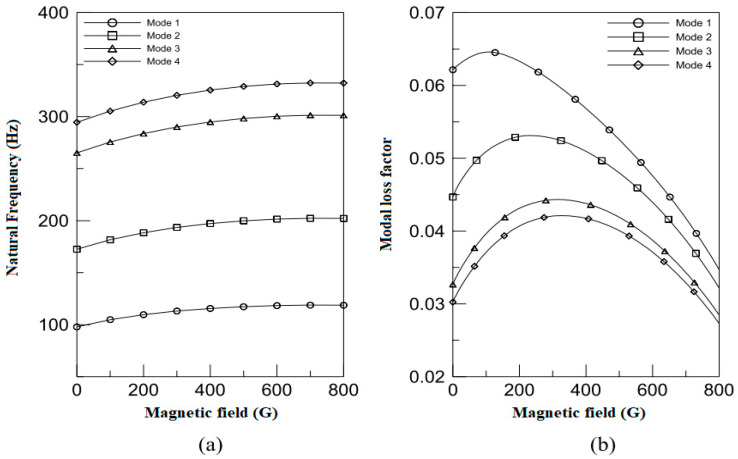
Effect of magnetic field on (**a**) natural frequency and (**b**) modal loss factor of the orthotropic sandwich plate system [102].

In comparison to MRE beam structures, few studies on sandwich shells and plates with an MRE core layer have been reported. Yeh [101] used the classical laminated plate theory (CLPT) to study the damping characteristics of a rectangular sandwich plate with an MRE core. The stiffness and modal loss factor were found controlled by fluctuating the intensity of magnetic field and MRE core layer thickness. Orthotropic sandwich plate dynamics with an MRE core and a restricting layer was studied by Yeh [102]. Figure 13 shows that increasing magnetic field intensity enhances natural frequencies at all modes while lowering modal loss factors at higher modes, as indicated in the FEM calculations.

After investigating the MRE sandwich plate under forced vibration, Aguib et al. [76] used computational and experimental approaches to examine its vibration response. It was shown that increments up to 0.5 T in magnetic fields increased the damping characteristics while decreasing vibration amplitude by 52%. Mikhasev et al. [103] carried out a theoretical investigation of the laminated MRE sandwich cylindrical shell structure′s eigen modes. It was discovered that the damping of the MRE sandwich shell increased as the magnetic field was amplified. Yeh [104] investigated the vibration properties of a sandwich cylindrical shell with an MRE core and a constraining layer. They used CLPT in the FE formulation to derive the governing equation of motion for a sandwich cylindrical shell. It was discovered that raising the magnetic field strength raises the natural frequency while lowering the modal loss factors, and an increase in modal loss factor and decrement in the natural frequency were also discovered by increasing the thickness of the MR layer.

Fewer studies are available on the application of the damping effect of an MRE; for example, Ulicny and Golden and Ottaviani et al. [105,106] suggested an MRE-based releasable fastener system that enables regulated joint release or separation in a shear or pull-off direction. When the fastener is engaged, the system delivers active or changeable dampening qualities. Additionally, MRE was considered for use in the construction of soft actuators by Bose et al. [107]. This mechanism ensures that airflow through the adjustable valve is maintained. In the MRE actuator, when exposed to a magnetic field, the MRE ring expands radially and forms a closable gap between the yoke and the ring. The magnetic field can be used to control the flow of air via a closable gap. Kashima et al. [108] presented another isotropic MRE-based soft actuator. As with other MRE actuators, this isotropic MRE actuator changes shape when a magnetic field is applied and returns to its original shape when the magnetic field is removed.

### 5.3. MRE Sandwich Structures Dynamic Responses under Different Parameters 

Apart from the applied magnetic field, additional parameters, such as the material and layer thickness of the face layers, the layer thickness of the core layer, boundary conditions, external disturbances, modes of vibration, and temperature, may have an impact on the natural frequencies, loss factors, and vibration amplitude of the MRE-based sandwich structures. Some of the related studies are mentioned below.

**Figure 14:**
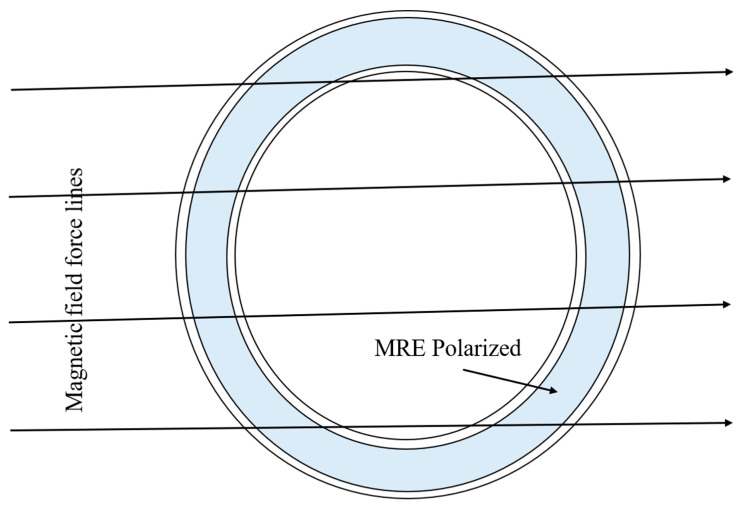
Three-layered circular cylindrical shell with polarized MRE in the magnetic field.

**Figure 15:**
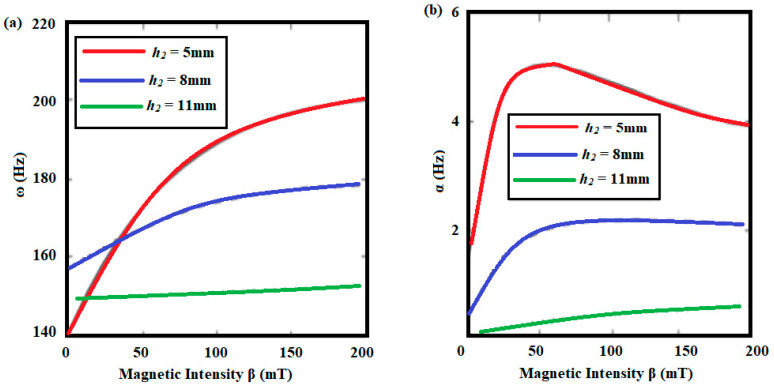
(**a**) Natural frequency ω and (**b**) damping ratio α vs. magnetic intensity β for various values of MRE thickness.

Mikhasev et al. [109] investigated magnetic fields′ effect on the free and forced vibrations of laminated cylindrical shells comprising a variety of MREs. Figure 14 and Figure 15 show the effect of the magnetic field, opening angles, and thickness ratios on the amplitude-frequency features of sandwich cylindrical shells.

**Figure 16:**
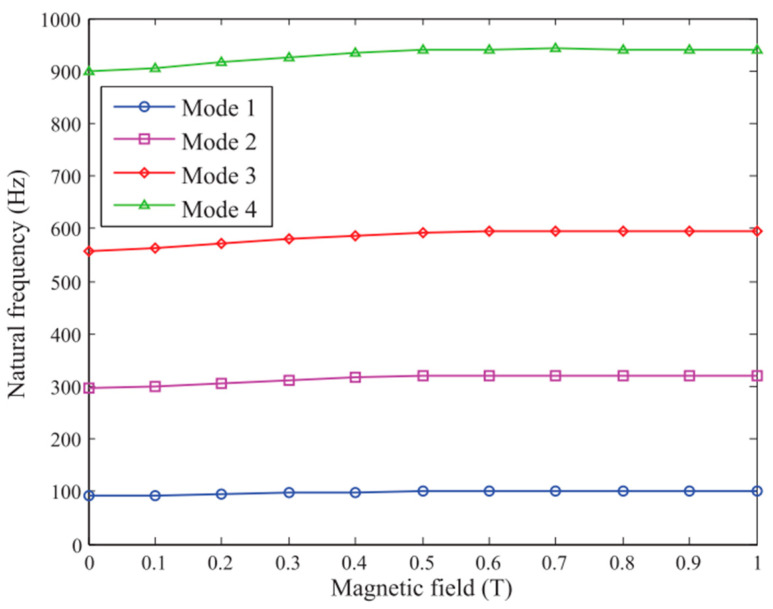
Natural frequency response under different magnetic fields [112].

The free vibrations of a rotating MR doubly tapered sandwich beam were investigated by Navazzi et al. [110], who presented the effects of the taper ratio, induced rotational speed, setting angle, and magnetic field intensity. Bornassi et al. [111] conducted a theoretical analysis of the free vibration of a rotating MRE sandwich beam in the sideways direction, taking into account the effects of core thickness, rotational speed, setting angle, hub radius, and applied magnetic field intensity. They also looked into the torsional vibrations of the tapered MRE sandwich beam while it was rotating. [112] The effect of magnetic field on natural frequencies for the first four modes on the non-rotating uniform cross section beam is shown in Figure 16.

Vemuluri and Rajamohan [113] used the composite laminated plate theory (CLPT) in FE formulation to derive the governing equations of motion for tapered composite MRE sandwich plates. In order to verify the effectiveness of the FE formulation, experimental measures and comparison with previous results found by Ramamoorthy et al. were used. With the effects of an applied magnetic field, face layer ply orientation, taper angle, aspect ratios, and the stiffness and loss factors of tapered MRE sandwich structures may be modified, according to the parametric studies.

Nanoparticles are currently gaining popularity among researchers due to their high rigidity, low density aspect ratio, and damping characteristics. MR materials can benefit from the addition of nanoparticles or fillers, which boost their magnetic resonance effects, sedimentation stability, stiffness, and damping. Some scientists studied the magnetic resonance impacts of nanoparticles augmented with MR materials to see how they behaved. However, adjusting the quantity and size of nanoparticles in MR materials yielded the greatest effects. Nanoparticles have recently been shown to improve the mechanical and dynamic behavior of MR materials, according to new study. Chena et al. [114] examined the mechanical behavior of MRE infused with several weight ratios of (0–7%) carbon black. Due to weak bonding between MRE and carbon, adding carbon black to MRE enhances the MR effect and tensile strength while reducing the damping ratio. Using black carbon as an injectant, Nayak et al. [115] investigated the mechanical behavior of MRE. Adding carbon black to MRE was found to boost the MR effect, improve tensile strength, and decrease the damping ratio because of the poor bonding between MRE and carbon.

The vibration responses for MRE sandwich beam composed of composite face sheets reinforced with functionally graded carbon nano tubes (CNT) were numerically analyzed by Fadaee and Talebitooti [116] using DQM. CNT distribution patterns with varying thicknesses were investigated. The natural frequencies and loss factors of sandwich beams were investigated in relation to the effects of magnetic fields, boundary conditions, CNT distributions and MRE core thickness. Using magnetoelastic loads, the dynamic behavior of the MRE-based sandwich beam with elastic face layers was investigated by Rokn Abadi et al. [117]. For various boundary conditions, the effect of magnetoelastic loads became more noticeable with higher magnetic fields and beam length.

**Figure 17:**
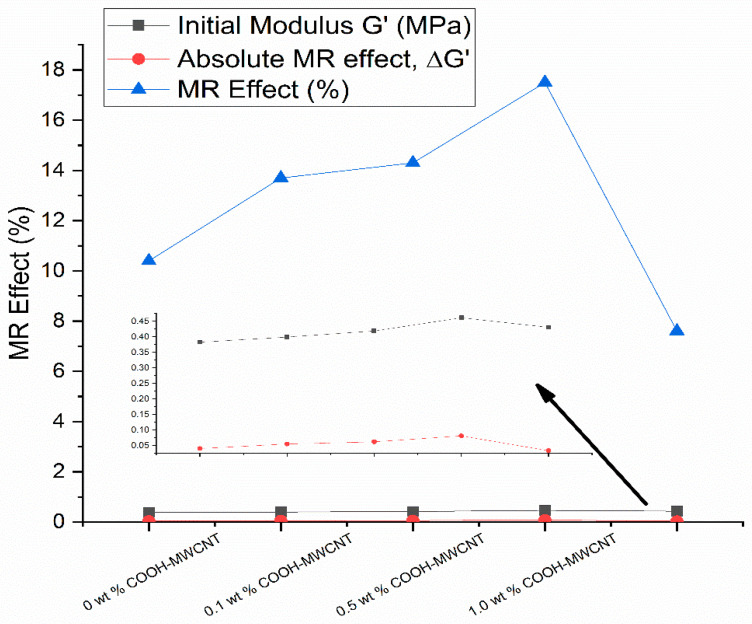
Viscoelastic properties of the MRE and MWCNT-MRE under applied magnetic fields. (**a**) Storage modulus (G′) of MRE and MWCNT-MRE. (**b**) Loss factor (Tanδ) of MRE and MWCNT-MRE.

The rheological characteristics of anisotropic MREs mixed with three types of carbon black (N330, N660, and N990) were investigated by Lu et al. [118]. The results show that the carbon black N990 sample outperforms the other samples in terms of MR effect and damping. Furthermore, as the carbon black N990 content increased, so did the MR properties. Sun and Li [119] studied the rheological properties of multi-walled carbon nano tubes (MWCNTs) reinforced with MRE by reinforcing MR samples with RTV rubber and 1% weight of MWCNT and 20% volume of iron particles. The results revealed that at zero and the increasing intensity of magnetic field, the MR nanocomposites demonstrated the absolute MR effect. They also tested the mechanical performance of MREs reinforced with MWCNT 0–3.5% weight and iron powder of 10% and 20% weight [120]. There was a significant improvement in stiffness and damping performance when MWNTs were added to MRE. There are significant differences between normal MRE and MWNT-reinforced MRE in terms of storage modulus as well as loss factor at zero magnetic field. Aziz et al. [121] investigated the viscoelastic characteristics of various types of MWCNT mixed with natural rubber-based MRE (0.1% weight). In terms of MR performance, MREs combined with COOH and OH-MWCNT outperformed pure MWCNT. Aziz et al. [122] conducted mechanical, morphological, thermal, and rheological experiments on MWCNT reinforced with natural rubber-based MRE. The experiment findings show that adding CNT to MRE enhances tensile strength by up to 11% while lowering heat stability, owing to surface flaws in MWCNT. In terms of rheological characteristics, MRE with CNT has a higher storage modulus than normal MRE, and the loss factor of MRE with CNT increases as the magnetic field increases. Aziz et al. [123] also examined the viscoelastic characteristics of MWCNT-reinforced MREs using 0–1.5 wt% MWCNT. When 1 wt% CNT was added to the MRE, the stiffness rose considerably, as seen in Figure 17.

**Figure 18:**
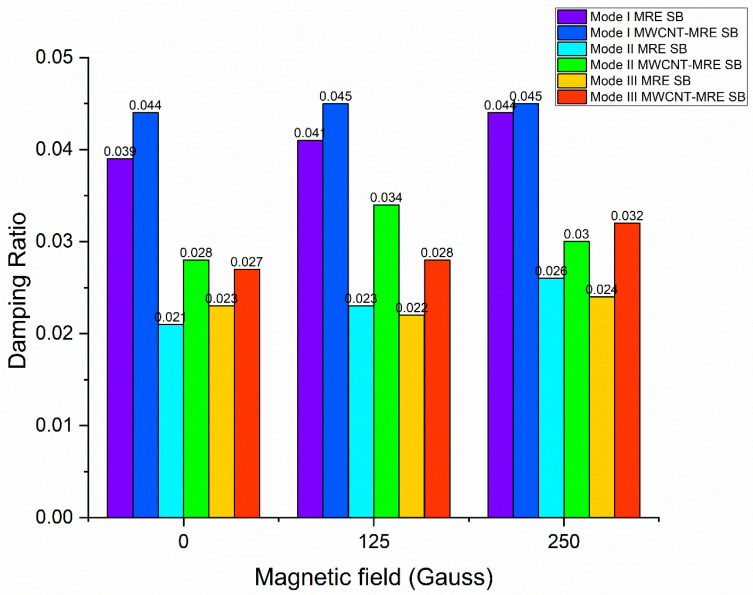
Effect of magnetic field on damping ratio.

**Figure 19:**
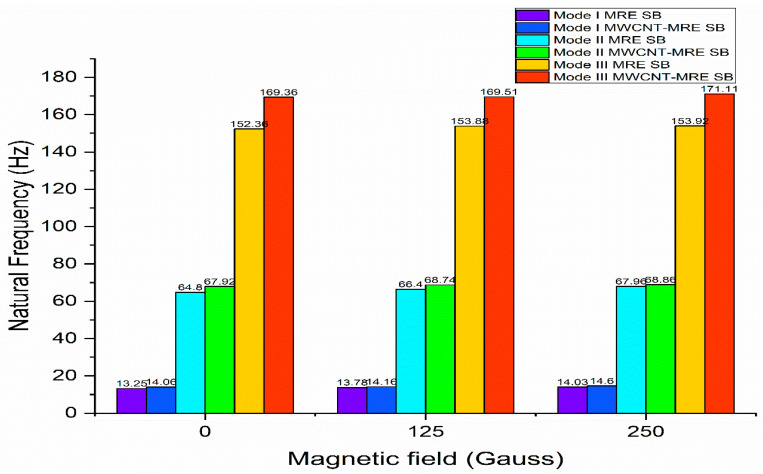
Effect of magnetic field on natural frequency.

To compare the viscoelastic characteristics of MRE with and without MWCNT reinforcement, Selvaraj and Ramamoorthy [124] utilized the ASTM756-05 standard test. They also used experimental and FEM approaches to study the free vibration characteristics of MRE and MWCNT-MRE sandwich beams. Figure 18 and Figure 19 indicate that the inclusion of MWCNTs in MRE enhanced the structure′s damping properties as well as its stiffness. The addition of carbon black fillers to MRE enhances the bonding with the elastomer matrix, resulting in the improved viscoelastic characteristics of MRE materials and an improved MR effect. In contrast, adding CNTs to the MRE enhances the storage modulus, loss factor, and MR effect. Additionally, the CNTs filled in the micron-sized gaps between the CIP enhanced the matrix′s interaction with them. With and without magnetic field enhancements, the rheological features of MRE materials have an impact on the matrix properties and interfaces between them.

### 5.4. Non-Linear Behavior of MRE Sandwich Structures

**Figure 20:**
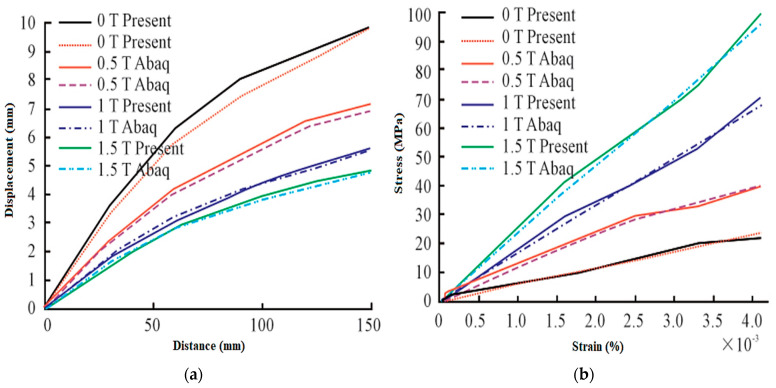
Stiffness and the damping characteristics of the sandwich beams. (**a**) Displacements Vs magnetic field intensities and (**b**) Stress strain curves.

Nonlinear behaviors in MRE sandwich structures have been observed, despite the fact that previous research indicated that MRE sandwich structures had a core operating in the pre-yield zone. The first-order natural frequency of a geometrically flawed MRE sandwich rose in response to an increasing external magnetic field [125]. Aguib observed the nonlinear static behavior of MRE sandwich beams made of composite materials. When the magnetic field was altered, the beam′s stiffness and damping properties changed. The strains and stresses rose in direct proportion to the magnetic field strength. As the magnetic field rose, beam bending reduced, as illustrated in Figure 20 [126].

### 5.5. Dynamic Characteristics and Optimum Design of Partially Treated MRE Sandwich Structures

A study of the literature shows that the stiffness and damping characteristics of sandwich structures fully treated with the MRE layer increase significantly as the applied field rises. For their part, the MRE-layered sandwich structures are heavier due to their elastomer′s high weight density. Applying the same magnetic field throughout a whole structure is also a difficulty. In order to obtain extreme controllability with a relatively slight treatment area and minimal energy consumption, partial MRE treatments are highly desired, especially when administered in the most optimum places. MRE partial segments are used in the core layer of sandwich structures that have been partially treated.

Regarding the characteristics of partly treated MRE sandwich constructions, different elastomer and structural factors, such as core layer thickness and complex shear modulus, have a significant impact. Furthermore, partial treatments, both in number and position, have a major impact on structural dynamics. No matter what the design, the end circumstances, or the modes of vibration are, partial treatments result in lower natural frequencies of the sandwich structure than untreated ones. Since the fully treated structure has a larger mass, this is a result of that. It is generally accepted that raising the applied field increases natural frequencies.

According to popular belief, the loss factors of fully treated sandwich structures are generally higher than those of partially treated sandwich structures. This is due to the latter structure′s lower dissipated energy. However, under certain boundary conditions and geometries, some studies found that partially treated sandwich structures had a higher loss factor than fully treated sandwich structures.

The benefit of partially treated sandwich structures over fully treated sandwich structures is that they can achieve a suitable layout with minimal treatments while providing nearly the same concert as fully treated sandwich structures. The design of a partially treated MRE sandwich structure necessitates determining a suitable treatment formation that yields the structure′s extreme controllability and vibration clampdown capability in terms of stiffness and damping. Several optimization problems in MRE sandwich structures have been developed for this purpose.

**Figure 21:**
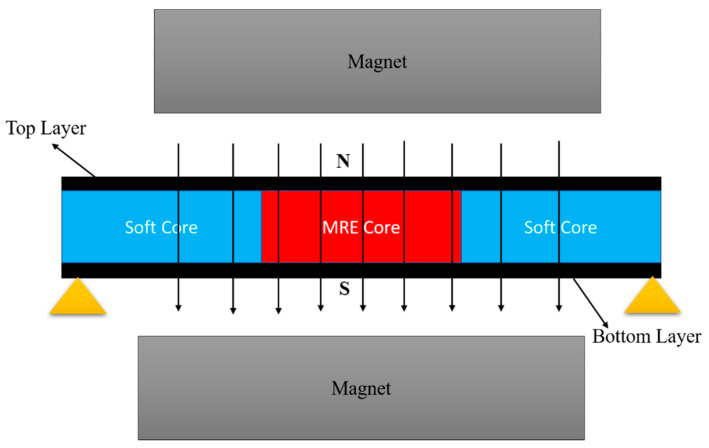
Representation of partially treated MRE sandwich beam.

Some numerical studies have been conducted to investigate the vibration characteristics of partially treated MRE sandwich structures. Zhou and Wang [127] investigated the MRE sandwich constructions that have been partially treated for vibrational properties. The numerical vibration responses of the sandwich beam with MRE and non-MRE sections were studied by Zhou and Wang [128]. The computational findings indicate that the sandwich beam′s flexural stiffness may be influenced by a magnetic field. Dwivedy et al. [129] statistically examined the instability areas on the sandwich beam with and without the MRE patch under periodic axial loads. A partially treated MRE sandwich beam in a magnetic field is shown in Figure 21. The application of the MRE patch and applied magnetic fields greatly enhanced the structural stability at varied boundary conditions.

**Figure 22:**
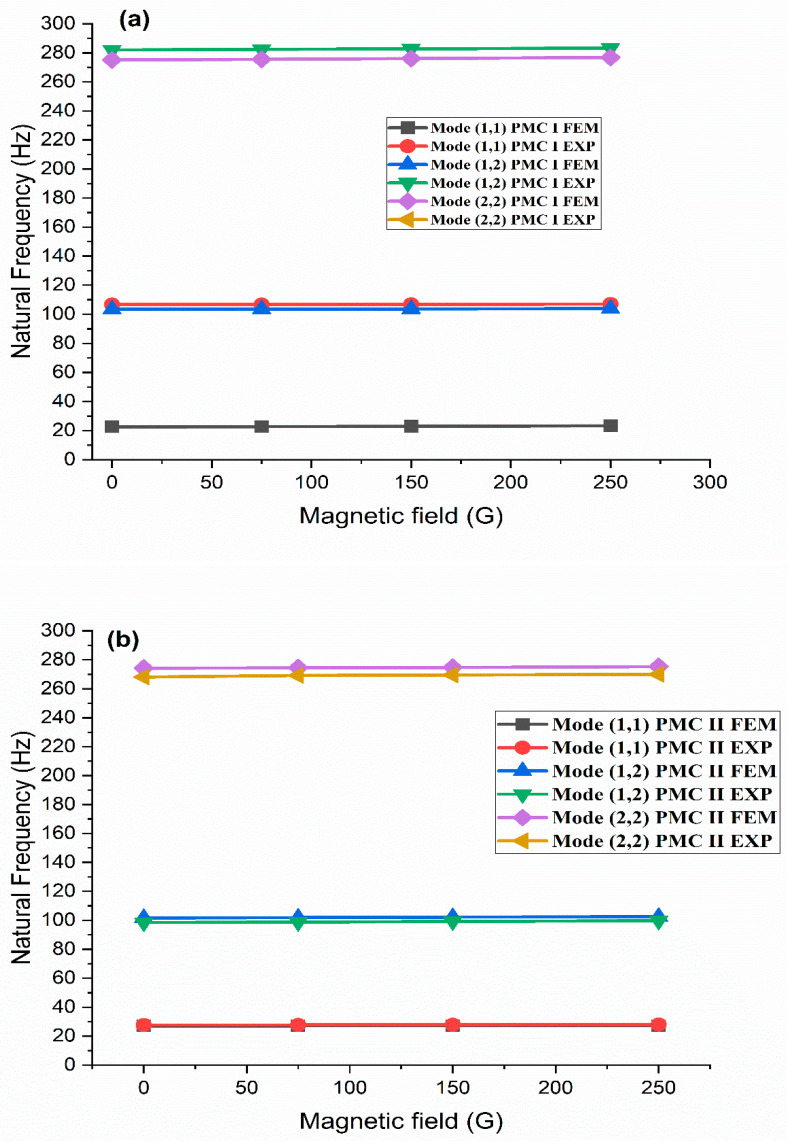

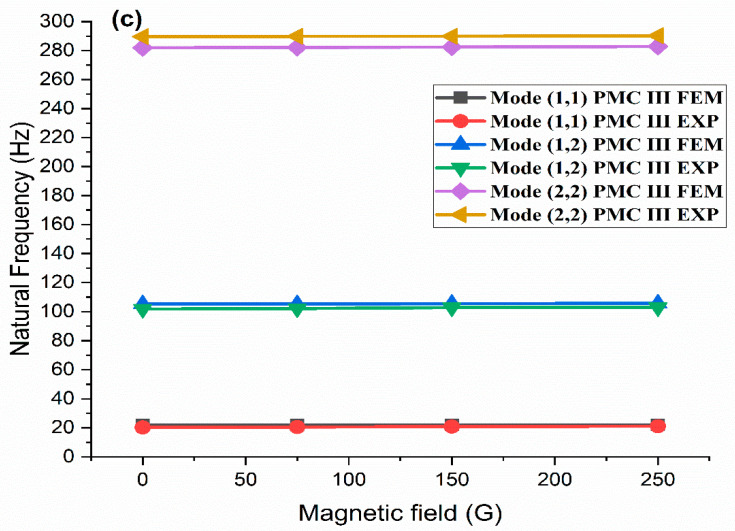
The effect of MRE size on the natural frequencies of different partially treated tapered composite sandwich configurations under different magnetic fields for (**a**) PMC I, (**b**) PMC II, and (**c**) PMC III.

Free vibration characteristics of a sandwich beam with conductive face sheets and a viscoelastic and MRE core were studied by Nayak et al. [130]. When compared to a viscoelastic-cored sandwich beam, the MRE sandwich beam reduced vibration by as much as 30%. They also found that increasing the quantity of MRE patches reduced vibration more effectively. The dynamic stability of different MRE sandwich beam designs was studied by Nayak et al. [75] using the FEM and harmonic balancing technique. They discovered that the MRE sandwich beam′s stability was impacted by applied magnetic fields. For tapered composite partly treated MRE sandwich plates, the governing equations of motion were derived using CLPT in FE formulation by Vemuluri et al. [131]. When the MRE pockets were divided into four sections, the peak natural frequencies increased. This was also observed with the tapered, partly treated MRE sandwiches. For all boundary conditions, a small change in the taper angle resulted in substantial variance in natural frequencies. The frequency response data are shown in Figure 22, which may be seen below. Partially treated MRE segments were utilized by Vemuluri et al. [132] in different tapered composite sandwich plates. From this, it can be deduced that the best sites for MRE treatment have a substantial influence on the dynamic characteristics under boundary conditions.

## 6. Conclusions

This review discusses the impact of several parameters on the dynamic responses of MRE-cored sandwich constructions, including the applied field, shape, material of the elastic layer, boundary conditions, excitation frequency, and external disturbances. Additionally, we reviewed mathematical models, manufacturing processes, experimentation methodologies, and solutions which yielded some amazing outcomes.

When sandwich structures with an MRE core layer are under influence of a magnetic field, the natural frequency, vibration amplitude, and the stiffness and damping characteristics of the structures can be dramatically altered. Furthermore, the increase in the tapering ratio with respect to width and thickness enhances natural frequencies while reducing loss factors in MRE cored sandwich structures, according to the recent study. In addition, sandwich structures with carbon nanoparticle reinforcement in composite face layers were explored. As a result, the sandwich structures become stiffer and more dampened. In MR materials, adding nanoparticles or fillers improves sedimentation control and improves the rheological characteristics.

Partially treated MRE sandwich constructions can sometimes outperform fully treated ones in terms of damping characteristics while weighting less. By designing semi-active/active controllers for the structures, the performance of partially and completely treated structures may be enhanced.

The majority of current research examines the vibration properties of the MRE sandwich beam in a uniform magnetic field. However, research on the vibration properties of MRE sandwich beams in non-homogeneous magnetic fields is relatively rare. The application of the MRE sandwich beam structure, in particular, is in its infancy. This is incompatible with fully exploiting the MRE′s characteristics in order to develop the corresponding frequency damping device. As a result, future research on the application of MRE sandwich beams must be strengthened. Additionally, there is a dearth of work in the literature on the nonlinear behavior of MRE sandwich structures and numerical analysis of functionally graded (FG) composite face sheets with an MRE core layer, both of which require additional attention in the future.

## Figures and Tables

**Table 1:** Related parameters of four-parameter viscoelastic model.

Strain Amplitude	Magnetic Flux Density (mT)	k_1_/KPa	k_2_/KPa
10%	0	514.37	1.669
	110	557.18	1.930
	220	564.21	2.406
	330	578.16	2.916
	385	621.34	3.919
